# Hemodynamic Effects of Positive Airway Pressure: A Cardiologist’s Overview

**DOI:** 10.3390/jcdd12030097

**Published:** 2025-03-10

**Authors:** Anna Di Cristo, Andrea Segreti, Nardi Tetaj, Simone Pasquale Crispino, Emiliano Guerra, Emanuele Stirpe, Gian Paolo Ussia, Francesco Grigioni

**Affiliations:** 1 Cardiology Unit, Fondazione Policlinico Universitario Campus Bio-Medico, Via Alvaro del Portillo 200, 00128 Roma, Italy; anna.dicristo@unicampus.it (A.D.C.); nardi.tetaj@unicampus.it (N.T.); simone.crispino@unicampus.it (S.P.C.); g.ussia@policlinicocampus.it (G.P.U.); f.grigioni@policlinicocampus.it (F.G.); 2 Research Unit of Cardiovascular Science, Department of Medicine and Surgery, Università Campus Bio-Medico di Roma, Via Alvaro del Portillo 21, 00128 Roma, Italy; 3 Department of Movement, Human and Health Sciences, University of Rome “Foro Italico”, Piazza Lauro de Bosis 15, 00135 Roma, Italy; 4 Cardiology Division, Department of Biomedical, Metabolic and Neural Sciences, University of Modena and Reggio Emilia, Policlinico di Modena, Via del Pozzo 71, 41124 Modena, Italy; 5 Department of Respiratory Disease, Hospital of Bolzano (SABES-ASDAA), Via Lorenz Böhler 5, 39100 Bolzano, Italy; emanuele.stirpe@sabes.it

**Keywords:** acute respiratory failure (ARF), bilevel positive airway pressure (BiPAP), cardiogenic pulmonary edema (CPE), continuous positive airway pressure (CPAP), non-invasive ventilation (NIV), obesity hypoventilation syndrome (OHS), obstructive sleep apnea (OSA), positive airway pressure (PAP)

## Abstract

Positive airway pressure (PAP) therapy is widely used to manage both acute and chronic respiratory failure and plays an increasingly important role in cardiology, particularly in treating patients with respiratory comorbidities. PAP, including continuous positive airway pressure and noninvasive ventilation, significantly impacts hemodynamics by altering intrathoracic pressure, affecting preload, afterload, and stroke volume. These changes are crucial in conditions such as acute cardiogenic pulmonary edema, where PAP can enhance gas exchange, reduce the work of breathing, and improve cardiac output. PAP reduces the left ventricular afterload, which in turn increases stroke volume and myocardial contractility in patients with left-sided heart failure. However, the role of PAP in right ventricular function and its effects on venous return and cardiac output are critical in the cardiac intensive care setting. While PAP provides respiratory benefits, it must be used cautiously in patients with right heart failure or preload-dependent conditions to avoid adverse outcomes. Additionally, in recent years, the use of PAP has expanded in the treatment of severe obstructive sleep apnea and obesity hypoventilation syndrome, both of which significantly influence cardiovascular events and heart failure. This review provides an in-depth analysis of the hemodynamic effects of PAP in cardiovascular disease, focusing on its impact on ventricular function in both acute and chronic conditions. Evaluating clinical studies, guidelines, and recent advancements offers practical insights into the physiological mechanisms and key clinical considerations. Furthermore, this review aims to serve as a helpful guide for clinicians, assisting in decision-making processes where PAP therapy is applied.

## 1. Introduction

Positive airway pressure (PAP), which includes continuous positive airway pressure (CPAP) and non-invasive ventilation (NIV), most commonly bilevel positive airway pressure (BiPAP), is indispensable for managing both acute and chronic respiratory distress.

In cardiology units, in recent decades, there has been a significant increase in hospital admissions of patients with primary non-cardiac conditions, such as lung diseases, kidney diseases, and infections [1]. Consequently, it has become essential for cardiologists to develop a broader skill set beyond their traditional field, including expertise in non-invasive ventilation [2,3,4].

According to the 2021 ESC guidelines, non-invasive positive pressure ventilation should be considered for patients experiencing respiratory distress (respiratory rate >25 breaths/min, SpO_2_ <90%) and should be initiated promptly to reduce respiratory distress and the need for mechanical endotracheal intubation, with a class IIa recommendation and level of evidence A [5].

Therefore, this review aims to highlight the hemodynamic effects of PAP treatment, focusing on cardiac patients with and without comorbidities in both intensive care units and general wards.

### Physiologic Cardiorespiratory Interaction During Non-Invasive Ventilation

**Figure 1:**
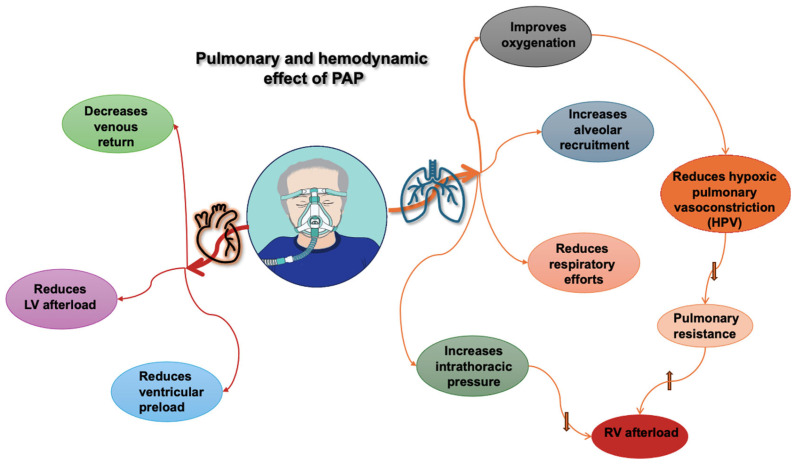
Pulmonary and hemodynamic effects of positive airway pressure (PAP). RV: right ventricle; LV: left ventricle, upward arrow indicates an increase, downward arrows indicate a decrease.

The interplay between the heart and lungs occurs due to their intrathoracic location and vascular interactions (See Figure 1). To understand this interaction, we should first clarify some definitions.

Cardiac output is the volume of blood ejected by the heart per minute. It is primarily determined by heart rate and stroke volume. A higher heart rate increases cardiac output directly while also increasing myocardial contraction, known as the Bowditch effect or staircase effect [6]. Conversely, stroke volume is determined by the preload, afterload, and contractility, which are closely interrelated [7].

Preload can be defined as the length of the myocardial sarcomere just before contraction, best approximated by end-diastolic volume, or as tension on the myocardial sarcomeres at end-diastolic pressure. Preload is related to venous return and right atrial pressure. The venous return depends on the difference between peripheral venous pressure and right atrial pressure (which corresponds to central venous pressure). According to Ohm’s law applied to fluid dynamics, it is inversely proportional to vascular resistance. In clinical practice, left ventricular preload is assessed by measuring pulmonary capillary wedge pressure.

Afterload is the resistance to ventricular ejection or the pressure the heart must generate to eject blood during ventricular contraction. Right ventricular afterload is commonly defined as pulmonary vascular resistance. Left ventricular afterload is proportional to aortic pressure and, therefore, to systolic blood pressure [8].

The well-known Frank–Starling curve explains the direct relationship between preload, afterload, and contractility, and thus cardiac output. This mechanism describes how an increase in ventricular end-diastolic volume, caused either by an increase in venous return (preload) or an increase in aortic resistance (afterload), is followed by an increase in the force of contraction [9].

On the other hand, intrathoracic pressure (ITP), defined as the pressure within the pleural cavity, is a significant determinant of preload and afterload due to its effect on transmural right atrial pressure and transmural pressure of the left ventricle (LV).

During spontaneous inspiration, the thoracic cavity actively expands as ITP decreases. This results in an increase in intrathoracic blood volume and venous return. The venous system is about 30 times more compliant than arteries and carries about 70% of the blood volume, with a large portion in the intra-abdominal cavity. As the diaphragm descends, intra-abdominal pressure increases, leading to compression of the venous system. Subsequently, the blood moves into the right atrium, further increasing venous return. In contrast, positive-pressure ventilation has the opposite effect. During inspiration, ITP increases due to passive lung expansion, resulting in a decrease in venous return and preload [10,11].

In respiratory distress, negative swings in ITP may cause negative right atrial pressure; however, the subsequent rise in venous return to the right ventricle is limited because the large venous vessels collapse as they enter the thorax. Further decreases in ITP will not increase venous return due to this flow limitation. However, there is no such limitation for the effects of negative swings in ITP on LV ejection [12].

In patients with airway obstruction (such as asthma or upper airway obstruction) or forced spontaneous inspiratory efforts, as seen in respiratory distress, negative swings in ITP may increase LV afterload, impeding LV ejection and promoting LV dilation. This can lead to LV failure, pulmonary venous congestion, and pulmonary edema, particularly if LV systolic function is already impaired. Therefore, removing negative ITP swings through positive-pressure ventilation reduces LV afterload without impeding venous return and improves alveolar oxygenation [13].

Hypoxic pulmonary vasoconstriction, also known as the Euler–Liljestrand mechanism, is a lung’s intrinsic mechanism for optimizing ventilation/perfusion matching and systemic oxygen delivery. It causes constriction of small intrapulmonary arteries in response to alveolar hypoxia, diverting blood to better-oxygenated lung segments. The vasoconstrictor response to hypoxia is unique to the pulmonary artery because systemic vasculature dilates in response to hypoxia, which also helps increase tissue oxygen delivery [14].

PAP is recommended in hospitalized patients with acute respiratory failure (ARF), including those with cardiogenic pulmonary edema, exacerbations of chronic obstructive pulmonary disease, and acute respiratory distress syndrome (ARDS). Both CPAP and BiPAP modes are safe and effective in patients with ARF. However, NIV BiPAP is preferred in cardiogenic pulmonary edema with hypercapnia, as it helps reverse hypercapnia and acidosis, reducing the need for intubation and thereby improving clinical outcomes [15,16].

## 2. Hemodynamic Effects of PAP in Cardiac Patients in the Intensive Care Unit

### 2.1. Impact on Congestion in Pulmonary Edema

In acute cardiogenic pulmonary edema (ACPE), the accumulation of interstitial fluid results in reduced pulmonary compliance, increased alveolar resistance, and compromised respiratory exchange. Edema reduces lung distensibility and promotes alveolar and small airway collapse. Inflammation-related mechanisms compromise air–blood barrier activity, and the presence of proteins and cellular debris hampers surfactant activity. The heightened surface tension at the small airway interface leads to atelectasis. Additionally, alveolar fluid increases airway resistance and mechanical compression in the peribronchial interstitium. The combined presence of reduced lung compliance and increased alveolar resistance raises the work of breathing, leading to respiratory failure and impaired gas exchange. Hypoxia-induced vasoconstriction in poorly ventilated alveoli leads to pulmonary hypertension, along with mechanical compression of the small vessels in the perivascular interstitium.

In ACPE, applying positive end-expiratory pressure (PEEP) keeps the airways open, counteracting alveolar collapse and improving gas exchange. Intra-alveolar resistance increases due to capillary compression. The connective tissue of intra-alveolar septa is in a continuum with the rest of the extracellular matrix of the lung; thus, extra-alveolar vascular resistance is reduced by elastic forces that stretch interstitial capillaries. Additionally, the patency of alveoli and small airways promotes blood oxygenation and counteracts hypoxic pulmonary vasoconstriction. However, if lung volume increases excessively, the opposite effect occurs: extra-alveolar resistance increases significantly, leading to an unfavorable outcome [11].

The early application of PAP (CPAP or BiPAP) in patients with ACPE improves acute respiratory failure and metabolic disturbance more quickly than conventional oxygen therapy. It may reduce the need for endotracheal intubation [17,18,19], although the effect on mortality reduction is controversial [20].

Compared with CPAP, BiPAP produces more significant improvements in oxygenation, carbon dioxide clearance, and breathing work in patients with pulmonary edema [21]. Detecting the B-lines pattern with a bedside lung ultrasound is a valid way to monitor the clearance of interstitial fluid and its distribution after the first hours of treatment in an emergency setting.

**Figure 2:**
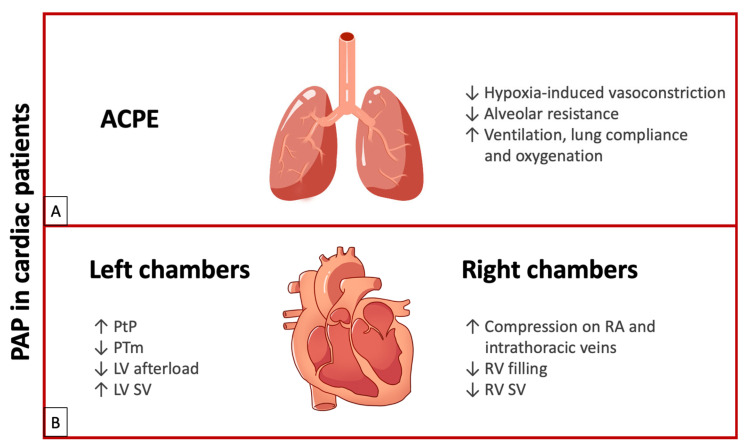
(**A**) Effects of PAP in ACPE. (**B**) Effects of PAP on left chambers and right chambers. ACPE: acute cardiogenic pulmonary edema; LV: left ventricle; PAP: positive airway pressure; PTm: transmural pressure; PtP: transpulmonary pressure; RA: right atrium; RV: right ventricle; SV: stroke volume; upward arrows indicate an increase; downward arrows indicate a decrease.

In summary, PAP use in patients with ACPE improves ventilation, lung compliance, and oxygenation, with the favorable outcome of reduced work of breathing, defined as the energy used by the respiratory muscles for ventilation (about 5% of VO2 max in resting condition). Reducing the right ventricular afterload due to decreased pulmonary vascular resistance improves right heart output. Finally, as transpulmonary pressure increases due to positive end-expiratory pressure, the left ventricular afterload is reduced and stroke volume increases (See Figure 2A).

### 2.2. Effect on Left Ventricular Function

In healthy individuals, a slightly increased workload of the healthy left ventricle can quickly compensate for the reduction in intrathoracic pressure during inspiration (which becomes more negative by about 1–2 mmHg). Hemodynamic consequences are minimal, unlike in a dysfunctional ventricle.

Two principal intrathoracic pressures must be considered: transmural pressure (PTm) and transpulmonary pressure (PtP). PtP is the difference between alveolar pressure and the pressure recorded in the pleural cavity. PTm is the pressure difference recorded inside and outside the cardiac chambers and large vessels. The transmural pressure of the left ventricle corresponds to the difference between the intracavitary LV pressure and transpulmonary pressure, which represents the ventricular afterload (See Table 1).

Generally, the application of PEEP induces an increase in alveolar pressure and, consequently, in PtP. Therefore, the difference between left intraventricular pressure and transpulmonary pressure decreases, reducing left ventricular transmural pressure. A reduction in PTm improves stroke volume due to decreased resistance (afterload) and increased myocardial contractility [11].

Cardiogenic shock and pulmonary edema are the most severe acute manifestations of heart failure, leading to high morbidity and mortality that significantly increase healthcare costs. Due to contractile dysfunction and pulmonary congestion, the hemodynamic deficit is often associated with respiratory disorders. Hypoxemia usually requires ventilatory support with supplemental oxygen up to NIV. BiPAP is preferable in the hospital setting, while CPAP is optimal in pre-hospital settings and low-equipped or extra-hospital emergencies [22]. Recommended candidates for PAP include patients with acute heart failure and systolic dysfunction, patients with acidosis (pH < 7.25), and patients not eligible for endotracheal intubation [22]. Patients with a higher likelihood of NIV failure (defined as the need for intubation) include those with cardiogenic shock and cardiac arrest, while elderly patients and those with comorbidities (multiple organ failure, frailty, poor mental status, weak cough, and high tidal volume on NIV) are less likely to experience failure [23].

In acute decompensated heart failure and functional mitral regurgitation (fMR), PAP therapy decreases PTm, thereby reducing fMR and improving stroke volume in patients with LV dysfunction [24,25].

In conclusion, the PAP effect on PtP and PTm leads to decreased resistance (LV afterload), enhanced myocardial contractility, and improved stroke volume in patients with LV dysfunction. Additionally, it reduces heart rate by moderating adrenergic hyperactivity through parasympathetic stimulation resulting from lung inflation. Furthermore, PAP increases respiratory flow, enhances alveolar ventilation, reduces lung atelectasis, improves oxygenation, reduces fMR, and decreases the work of breathing (See Figure 2B).

### 2.3. Effect on Right Ventricular Function

The right ventricle (RV) is more susceptible to increased intracavitary (and transmural) pressure and systemic hypotension. It adapts well to preload variation but responds poorly to rapid pressure changes [26].

The increase in PtP induced by PEEP results in a compressive effect on the right atrium, increasing right atrial pressure and decreasing venous return. Additionally, the rise in PtP can compress intrathoracic veins (superior and inferior vena cava), increasing resistance to venous return. Consequently, compromised RV filling reduces stroke volume. Elevated PEEP increases tricuspid regurgitation due to pulmonary hypertension and right ventricular overloading [27,28,29]. Less significantly, lowering the diaphragm due to increased lung volume after PEEP increases intra-abdominal pressure, peripheral venous pressure, venous return, stroke volume, and systemic arterial pressure [11].

In preload-dependent conditions (such as marked hypotension, pericardial effusion, or pulmonary embolism), slight variations in volume status significantly affect cardiac output. Venous filling becomes crucial for optimal ejection. These vulnerable patients are most susceptible to the effects of PEEP. Patients with right atrial pressure below 10 mmHg will experience a more marked reduction in venous return with PEEP application, leading to an unfavorable hemodynamic outcome [11]. 

In patients with acute tamponade or pre-tamponade pericardial effusion, the right heart shows limited adaptation to increased filling pressures caused by fluid accumulation in the pericardial sac. Compression of the right heart chambers reduces venous return. If this is associated with respiratory dysfunction, PEEP can further reduce venous return, collapsing cardiac output. In cases of hemodynamic instability and respiratory failure, invasive ventilatory support is preferred [30].

Similarly, in patients with marked hypotension, reduced venous return due to hypovolemia can be worsened by PEEP. Rapid volume filling is necessary to maintain volemia, ensure venous return, improve ventricular preload, and increase output [31].

Submassive or massive pulmonary embolism rapidly increases RV afterload, but RV hardly adapts to this condition. This leads to RV dilation, bowing of the interventricular septum into the LV, reduced LV filling, and obstructive shock. Hypoxia correction is often necessary, but PAP is not the first choice due to its effects on decreasing RV preload in pre-existing hypotensive conditions and its limited impact on reducing RV afterload in cases of vascular obstruction [32]. 

In conclusion, the effect of PAP on the right heart remains controversial. PAP can reduce venous return, compromising RV filling and decreasing stroke volume. Therefore, PAP should be carefully managed in preload-dependent conditions, such as hypotension, pericardial effusion, or pulmonary embolism (See Figure 2B).

## 3. PAP in Cardiovascular Comorbidities

### 3.1. Obstructive Sleep Apnea

Obstructive sleep apnea (OSA) is a breathing disorder caused by the collapse of the pharyngeal airway during sleep. Multiple episodes of hypopnea (reduction in airflow) or apnea (interruption of airflow) affect quality of life, leading to fragmented sleep, daytime sleepiness, and impaired cognition.

Treatment with CPAP in patients with OSA improves sympathetic activity, reduces cardiac afterload, and lowers the risk of ventricular arrhythmias. NIV has shown even better results when compared to CPAP therapy [33]. 

A study examining RV function in patients with OSA showed a significant improvement in RV parameters after CPAP treatment, including restoration of RV wall contraction uniformity and a reduction in RV volume [34]. Long-term CPAP therapy (at least 3 months) improves endothelial function in patients with OSA, likely due to recovery from systemic hypoxia [35]. CPAP also reduces oxidative stress and increases plasma antioxidant status by enhancing nitric oxide metabolites [36]. 

Finally, CPAP treatment of severe OSA tends to lower the arousal threshold, reduce daytime sleepiness, and improve quality of life. CPAP administration in patients with heart failure improves systolic function, reduces myocardial energy expenditure, and decreases respiratory muscle workload [37]. 

### 3.2. Obesity Hypoventilation Syndrome

Obesity hypoventilation syndrome (OHS) is a growing concern due to the current obesity epidemic. OHS is defined by the combination of obesity (BMI ≥ 30 kg/m^2^), daytime hypercapnia (arterial CO_2_ tension ≥ 45 mmHg), and sleep-disordered breathing after ruling out other causes of alveolar hypoventilation [38]. 

OHS is often diagnosed by the presence of acute-on-chronic hypercapnic respiratory failure.

NIV treatment overcomes CPAP and lifestyle modification in improving pulmonary hypertension, left ventricular hypertrophy, and functional capacity, likely due to a reduction in pulmonary artery systolic pressure or improved respiratory function [39]. PAP treatment also improves PaCO_2_ and sleep parameters measured by polysomnography, reduces daytime sleepiness, and improves forced vital capacity (FVC), forced expiratory volume in 1 s (FEV1), and 6-min walk distance [40]. 

Although CPAP is more straightforward and less expensive, NIV provides better ventilatory support, especially in patients with non-obstructive phenotypes. Thus, if CPAP treatment fails (e.g., no improvement in gas exchange, clinical symptoms, or frequent hospitalizations for acute respiratory insufficiency), switching to NIV should be considered.

## 4. High-Flow Nasal Oxygen

### High-Flow Nasal Oxygen

High-flow nasal oxygen (HFNO) has become increasingly widespread in recent years. Current clinical guidelines recommend HFNO as the first-line treatment for acute respiratory failure [41]. 

HFNO delivers a controlled concentration of heated and humidified oxygen at high flow rates (up to 60–80 L/min) through a nasal cannula. It reduces reintubation rates and mortality in patients with acute respiratory failure [42]. HFNO generates a flow-dependent positive pressure effect (up to 8 cmH_2_O, especially with the mouth closed), increases tidal volume, and helps wash out CO_2_ by reducing dead space in the upper airway.

Thanks to its comfortable interface and humidification, HFNO enhances patient comfort and is better tolerated than non-invasive ventilation (NIV). HFNO can be a suitable alternative to non-invasive positive-pressure ventilation for patients with acute respiratory failure [43,44].

## 5. Complications of PAP Treatment

The success of PAP therapy depends on several factors, including appropriate patient selection, the patient–ventilator interface, staff expertise, and effective patient monitoring. PAP treatment has several contraindications, including a Glasgow Coma Scale score of ≤8, active and persistent vomiting, severe active upper gastrointestinal bleeding, facial or upper airway trauma/burns, and upper airway obstruction, as shown in Table 2.

**Figure 3:**
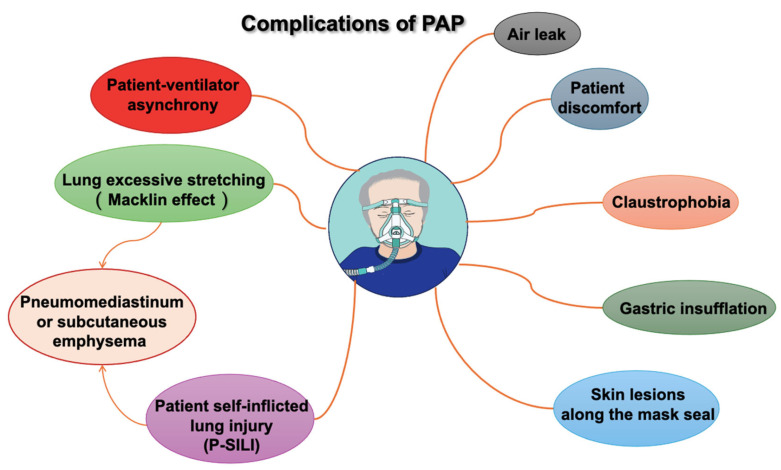
Complications of positive airway pressure (PAP).

The most frequent PAP complications, such as those associated with oronasal or full-face masks and helmets, are minor and typically related to the interface. These complications include air leaks, skin lesions along the mask seal (especially on the nose and forehead), gastric insufflation, claustrophobia, discomfort, and patient ventilator asynchrony, as shown in the Figure 3.

Alternating between oronasal and full-face masks is an effective strategy to improve comfort and tolerance. PAP should be discontinued promptly if the patient’s condition deteriorates, as indicated by worsening pH or respiratory rate. Light sedation, such as dexmedetomidine, can reduce the risk of delirium and discomfort associated with treatment [45].

In recent years, the concept of patient self-inflicted lung injury (P-SILI) has been proposed to describe lung damage in patients with spontaneous breathing and high respiratory drive. P-SILI is caused by intense inspiratory effort during acute respiratory failure and patient–ventilator asynchronies, often accompanied by increased respiratory drive and elevated transpulmonary pressure. If the CPAP or BiPAP NIV pressures are too low and the lungs are not sufficiently recruited and cannot be ventilated homogeneously, intrapulmonary traction and shear forces can occur, causing the corresponding damage. This can result in over-distension of ventilated areas during inspiration and increased pulmonary strain [46].

High tidal volumes and excessive lung stretching (e.g., during persistent coughing) can cause ruptures along the alveolar tree, allowing air to dissect toward the pulmonary hilum. This phenomenon, known as the Macklin effect, occurs mainly in ARDS patients and can lead to pneumomediastinum, subcutaneous emphysema, and, less frequently, pneumopericardium, pneumothorax, and air embolism [47,48].

On the other hand, inappropriately low tidal volumes can cause atelectasis, patient–ventilator desynchronization, and suboptimal gas exchange [49].

**Figure 4:**
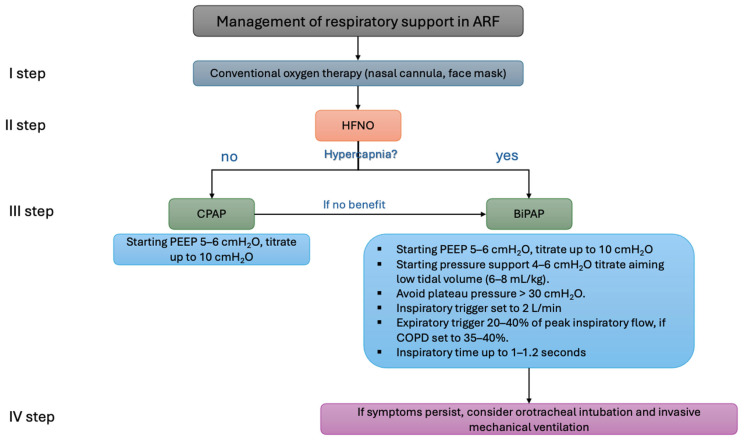
Example of the algorithm for oxygen support in patients with acute respiratory failure. ARF: acute respiratory failure; HFNO: high-flow nasal oxygen; CPAP: continuous positive airway pressure; BiPAP: bilevel positive airway pressure; PEEP: positive end-expiratory pressure; COPD, chronic obstructive pulmonary disease.

To reduce the patient’s inspiratory effort and the likelihood of PAP failure, lung-protective ventilation with low tidal volumes (6–8 mL/kg of predicted body weight) is recommended, as shown in the Figure 4. Tidal volumes exceeding 9.5 mL/kg are associated with PAP failure. If symptoms such as a high respiratory rate or hypoxemia persist despite positive airway pressure therapy, endotracheal intubation should be considered [50].

PEEP levels should be titrated to 5–8 cmH_2_O (up to a recommended maximum of 10 cmH_2_O) to facilitate effective triggering. Pressure support should be set to 6–8 cmH_2_O, aiming for a plateau pressure below 30 cmH_2_O while avoiding high tidal volumes (>9.5 mL/kg). Inspiratory and expiratory triggers should be optimized for patient–ventilator synchronization. Rise time should be adjusted to ensure patient comfort, with the ideal inspiratory time set at 1–1.2 s [51,52].

## 6. Conclusions

PAP, encompassing CPAP and BiPAP modalities, is a cornerstone of respiratory distress treatment in acute and chronic settings. In cardiovascular patients, its primary application is in managing acute cardiogenic pulmonary edema.

The hemodynamic effects of PAP are varied and complex, impacting left and right ventricular function and depending on the patient’s volume status.

PAP enhances pulmonary gas exchange, reduces breathing workload, and improves cardiac output by lowering the left ventricular afterload and right ventricular preload.

However, high positive airway pressure values increase pulmonary vascular resistance, raise the right ventricular afterload, and decrease left ventricular filling. As a result, PAP should be used with caution in patients with hypotension and right heart failure.

In patients with obstructive sleep apnea and obesity hypoventilation syndrome, PAP treatment improves quality of life, reduces myocardial energy expenditure, and decreases respiratory muscle workload.

The success of PAP treatment depends on several factors, including appropriate patient selection, the patient–ventilator interface, staff competence, and effective patient monitoring. Most complications are minor, but without proper precautions or protective ventilation strategies, more serious conditions like patient self-inflicted lung injury (P-SILI) or the Macklin effect can occur.

## Figures and Tables

**Table 1:** Main pathophysiological determinants of cardio-respiratory interaction during PAP.

Preload	The load imposed on myocytes before contraction, better defined as the tension on myocardial sarcomeres at end-diastole
Afterload	Resistance to ventricular ejection, or the amount of pressure the heart must generate during ventricular contraction
Cardiac output	The volume of blood ejected by the heart per minute
Intra-alveolar resistance	Vascular resistances depending on alveolar capillaries
Extra-alveolar vascular resistance	Vascular resistances depending on the vessels of the alveolar septa
Intra-thoracic pressure (ITP)	Pressure within the pleural cavity
Transpulmonary pressure (PtP)	Difference between alveolar pressure and pressure recorded in the pleural space
Transmural pressure (PTm)	Pressure differences recorded inside and outside the cardiac chambers and large vessels, representing ventricular afterload
Venous return	Blood flow from the periphery to the right atrium, depending on the difference between peripheral venous pressure and right atrial pressure

**Table 2:** Contraindications of noninvasive ventilation.

**Absolute contraindications**
Glasgow Coma Scale ≤8
Active and persistent vomiting or severe active upper gastrointestinal bleeding
Facial or upper airway trauma/burns
Upper airway obstruction
**Relative contraindications**
Hemodynamic instability
Facial anatomic anomalies
Severe upper gastrointestinal bleeding
Impaired airway protective reflexes, such as coughing and swallowing
Bowel obstruction
Uncooperative patient
Abundant respiratory secretions
Recent facial, upper airway, or upper GI tract surgery
Epistaxis
Undrained pneumothorax

## Data Availability

No new data were created or analyzed in this study.
